# Image-guided versus landmark-guided suprascapular nerve block for shoulder pain in rotator cuff tears: a systematic review

**DOI:** 10.1016/j.xrrt.2025.09.002

**Published:** 2025-09-17

**Authors:** Andrew Kailin Zhou, Dave Osinachukwu Duru, Saroop Nandra, Andrew Metcalfe, Salma Chaudhury

**Affiliations:** a University of Warwick, Coventry, UK; b Department of Trauma and Orthopaedics, University Hospital Coventry and Warwickshire, Coventry, UK; c University of Cambridge, Cambridge, UK; d Department of Trauma and Orthopaedics, Addenbrookes Major Trauma Unit, Cambridge University Hospitals, Cambridge, UK

**Keywords:** Suprascapular nerve block, Rotator cuff tears, Conservative management, Landmark guided, Ultrasound guided, Shoulder pain

## Abstract

**Background:**

Suprascapular nerve block (SSNB) is a recognized treatment for chronic shoulder pain, including pain from rotator cuff tears. While it is purported that image-guided SSNB improve injection accuracy over landmark-guided techniques, the impact on clinical outcomes remains unclear. This systematic review compared image-guided vs. landmark-guided SSNB in patients with rotator cuff tears, evaluating efficacy, pain relief, functional improvement, complications, and duration of relief.

**Methods:**

We searched PubMed, MEDLINE, Cochrane Library, Embase, and CINAHL (inception to April 2025) for prospective or retrospective studies comparing image-guided (ultrasound, fluoroscopy, computed tomography, or arthroscopic) to landmark-guided SSNB. Two reviewers independently screened titles/abstracts and full texts, with discrepancies resolved by consensus. Data on pain outcomes, functional scores, complications, and duration of pain relief were extracted. Risk of bias was assessed for each study.

**Results:**

Thirty studies were included, comprising 25 randomized controlled trials, 2 nonrandomized prospective studies, and 3 retrospective studies, totaling 2,205 patients. Both image-guided and landmark-guided techniques significantly reduced pain and improved shoulder function, with pain reduction typically ranging from 3.2 to 5.5 points on a 0-10 visual analog scale at 48 hours postoperatively. There was no consistent evidence indicating superior clinical outcomes with image-guided techniques in terms of pain relief, functional improvement, complication rates, or duration of analgesia.

**Conclusion:**

Both image-guided and landmark-guided SSNB techniques provide effective pain relief and functional improvement in patients with rotator cuff-related shoulder pain. Despite potential procedural advantages of image guidance, such as reduced needle repositioning and higher first-attempt success rates, these benefits did not translate into consistently superior clinical outcomes. This systematic review suggests landmark-guided SSNB offer similar outcomes to image-guided techniques, with implications for resource and expertise availability.

Rotator cuff tears are a common cause of chronic shoulder pain and dysfunction, reducing quality of life.^14^ While conservative treatments such as rest or physiotherapy are typically the first line of management, persistent or refractory pain may necessitate interventional approaches.^14^ Among these, suprascapular nerve block (SSNB) is a well-established technique for shoulder analgesia. There has been increasing interest in SSNB in place of glenohumeral joint injections, given the suprascapular nerve provides approximately 70% of sensory innervation to the shoulder joint.^14^ SSNB has demonstrated efficacy in reducing pain, improving range of motion, and facilitating rehabilitation in patients with rotator cuff pathology and other shoulder conditions.^14^

Traditionally, SSNB was performed using a landmark-guided technique, relying on surface landmarks such as the acromion and scapular spine to locate the nerve at the suprascapular notch or within the supraspinous fossa.^7^^,^^14^ However, this method may be limited by individual anatomical variability.^36^^,^^41^ Inaccurate needle placement may lead to suboptimal analgesia and complications such as pneumothorax or neurovascular injury; though these are uncommon in experienced hands.^20^

With increased availability of clinical imaging modalities, image-guided SSNB has gained popularity, particularly ultrasound-guided injection, as it is relatively easy to use and traditionally the cheapest form of image guidance.^26^ Ultrasound allows visualization of the suprascapular notch, facilitating accurate needle placement while avoiding adjacent structures.^37^ Fluoroscopy and computed tomography (CT) guidance are alternative image-guided methods for localizing the suprascapular notch.^35^ Image guidance is thought to improve clinical outcomes and reduce risk of complications via increased accuracy. One study reported a 92.5% accuracy rate in targeting the suprascapular nerve with ultrasound guidance, compared to 72.5% with landmark-based techniques.^31^

Despite these theoretical benefits, it remains uncertain whether image guidance translates to significantly improved patient outcomes compared to landmark-guided SSNB. Although several studies have examined SSNB in the context of shoulder pain, there has been no systematic reviews focused specifically on the rotator cuff tear subgroup. Due to the age-related degenerative etiology and high-operative failure rates associated with larger tears, SSNB is an important treatment modality. Given the procedural and cost implications of requiring imaging for every SSNB, it is important to establish if the added complexity of image guidance provides a meaningful benefit to patients, warranting a focused systematic review.

## Methods

This review was conducted in accordance with the Preferred Reporting Items for Systematic Reviews and Meta-Analyses guidelines. The methodology was registered to International Prospective Register of Systematic Reviews (CRD420251027141).

### Inclusion and exclusion criteria

We included all clinical studies (randomized or nonrandomized, prospective or retrospective) that compared an image-guided suprascapular nerve injection to a landmark-guided suprascapular nerve injection in patients with rotator cuff tear. We have also included patients who had undergone rotator cuff surgery, with SSNB used for postoperative pain relief. Given the limited literature specifically pertaining to rotator cuff tears, we also included studies with heterogenous shoulder pain populations (eg, adhesive capsulitis, subacromial impingement, and acromioclavicular joint pathology) if a majority had rotator cuff pathology. Both published peer-reviewed articles and relevant abstracts were considered, with no date restrictions. We excluded cadaveric studies, case reports/series, and conference abstracts lacking sufficient data.

The interventions of interest were:

1) Image-guided SSNB: defined as SSNB performed with real-time imaging guidance (ultrasound, fluoroscopy, CT, or arthroscopic guidance).
2) Landmark-guided SSNB: defined as SSNB performed using only anatomical landmarks for needle placement (no real-time imaging).

Studies had to report at least one of the outcomes of interest: pain intensity (eg, visual analog scale [VAS]), shoulder function scores (eg, Shoulder Pain and Disability Index (SPADI); range of motion; Constant–Murley Score), complications, and/or duration of pain relief. If multiple follow-up time points were reported, we extracted the longest follow-up for outcome comparisons, as well as earlier time points for descriptive analysis. For trials with multiple intervention arms, we included only the arms relevant to our comparison.

### Search strategy

A comprehensive search was conducted across 5 databases: PubMed, MEDLINE, Embase, Cochrane Library (CENTRAL), and CINAHL. The search spanned from each database's inception to April 2025. We used combinations of keywords and Medical Subject Headings terms for “suprascapular nerve block” or “suprascapular nerve injection,” and terms for “ultrasound” or “image-guided” vs. “landmark” or “blind” injection, and “rotator cuff tear” or “shoulder pain.” An example of our search strategy was: (Suprascapular OR "suprascapular nerve") AND (block OR injection) AND (ultrasound OR ultrasonography OR image-guided OR guided OR landmark OR blind) AND (shoulder OR rotator cuff OR shoulder pain).

We also searched reference lists of relevant articles and prior reviews to identify additional studies. No date limits were applied, and only English articles were included.

**Figure 1:**
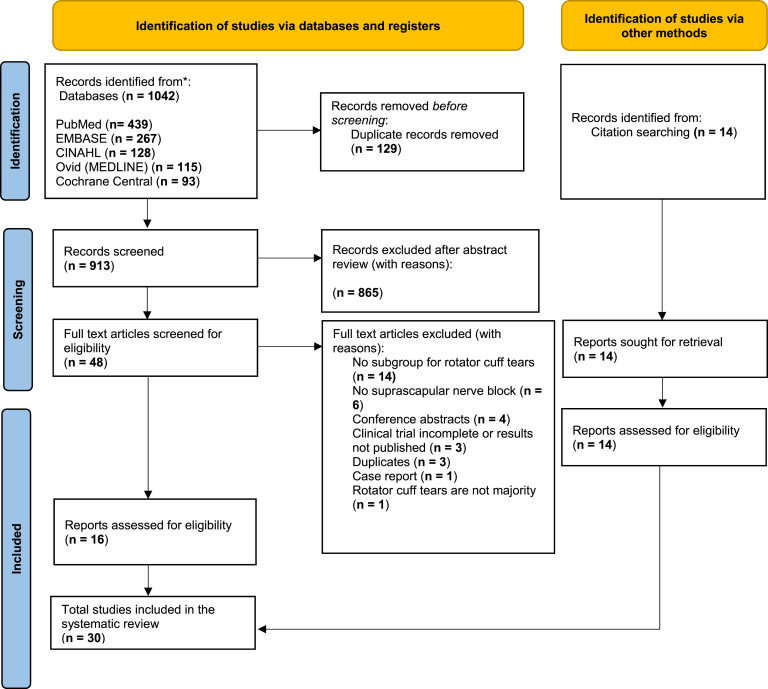
PRISMA flow diagram of the study selection process for the systematic review. *PRISMA*, Preferred Reporting Items for Systematic Reviews and Meta-Analyses.

All search results were imported into a Rayyan (Rayyan AI, Cambridge, MA, USA) and duplicates were removed. Two reviewers independently screened titles and abstracts against the inclusion criteria. Studies marked as “include” or “unclear” by either reviewer underwent full-text review. The same 2 reviewers then examined full-text articles to determine final inclusion, with reasons documented for exclusions. Any disagreements were resolved through discussion or consulting a third reviewer. A Preferred Reporting Items for Systematic Reviews and Meta-Analyses flow diagram summarizing the study selection process was prepared (Fig. 1).

### Data extraction

Data were extracted independently by 2 reviewers using a standardized form. From each included study, we collected: author(s), year, study design (randomized controlled trial [RCT], cohort, retrospective), sample size (number of patients or shoulders), patient population (specific shoulder pathology, eg, rotator cuff tear, frozen shoulder, arthritis), operation details, details of the intervention (image guidance modality) and control (landmark technique details), and follow-up duration. For outcomes, we extracted pain scores (eg mean VAS or Numeric Rating Scale scores at baseline and follow-ups for each group), functional outcomes (eg, SPADI, Constant–Murley score, or range of motion), and any reported complications. When multiple time points were reported, we recorded outcomes at baseline and at the final follow-up post-injection and earlier time points for trajectory description if provided. If outcome data were only presented in graphs, we approximated values using plot digitization when possible. Authors were contacted for additional data.

Two reviewers independently assessed the risk of bias (RoB) of each included study using Cochrane RoB 2.0 for RCTs and Risk Of Bias In Nonrandomized Studies (ROBINS)-I tool for nonrandomized studies. Discrepancies were resolved by consensus.

## Results

Our database search yielded 913 records after removing duplicates, of which 18 studies fulfilled the inclusion criteria (Fig. 1). Twelve additional studies were identified through forward and backward citation searching beyond the original search window. Table I provides a summary of the included studies. The included studies comprised 25 RCTs, 2 nonrandomized prospective studies, and 3 retrospective studies (Table I).

**Table I:** Summary of study characteristics and patient demographics (nonoperative SSNB above + postoperative SSNB below).

Reference	Study type	Total patients (shoulders)	Country	Type of SSNB	Number of SSNB (n)	Mean age	Female subjects, n (%)	Rotator cuff tear diagnosis criteria
Nonoperative SSNB studies (5)								
Coory et al, 2019^6^	Single-center RCT	42 (43)	Australia	US-guided SSNB	21	70	7 (33.3)	US and MRI
Shanahan et al, 2003^34^	Multi-center RCT	83 (108)	Australia	Landmark-guided SSNB	56	73	26 (46.4)	US
Shanahan et al, 2004^35^	Single-center RCT	67 (77)	Australia	(1) Landmark-guided SSNB (2) CT-guided SSNB	(1) 40 (2) 37	(1) 74.5 (2) 76	(1) 19 (47.5) (2) 20 (54.1)	US
Vecchio et al, 1993^40^	Single-center RCT	28	UK	Landmark-guided SSNB	15	59.9	7 (46.7)	Arthrography
Yıldızhan et al, 2024^43^	Single-center RCT	80	Turkey	(1) US-guided SSNB at spinoglenoid notch (2) US-guided SSNB at suprascapular notch	(1) 40 (2) 40	(1) 57.1 (2) 57.2	(1) 29 (72.5) (2) 13 (32.5)	MRI and US
Postoperative SSNB studies (25)								
Aliste et al, 2018^1^	Single-center RCT	40	Chile	US-guided SSNB + infraclavicular block (ICB)	20	57.9	11 (55.0)	NA
Auyong et al, 2018^2^	Single-center RCT	189	USA	US-guided SSNB	63	55	21 (33.3)	N/A
Botros et al, 2023^4^	Single-center RCT	66	Egypt	US-guided SSNB + costoclavicular block	33	38	11 (33.3)	Clinical
Choi et al, 2020^5^	Single-center RCT	118	Korea	Landmark-based SSNB	59	55	34 (57.6)	Intraoperative
Desroches et al, 2016^8^	Single-center RCT	53	France	Landmark-guided SSNB	28	60.8	11 (39.3)	MRI
Dhir et al, 2016^9^	Single-center RCT	59	Canada	US-guided SSNB + ANB	29	46.5	7 (24.1)	Clinical
Doğan et al, 2022^10^	Single-center RCT	52	Turkey	US-guided SSNB	27	52.6	15 (55.6)	NA
Haciomeroglu et al, 2024^11^	Single-center PCS	50	Turkey	US-guided SSNB + subacromial injection (SAI)	25	57.5	21 (84)	MRI
Han et al, 2013^12^	Single-center RCT	70	Korea	Landmark-guided SSNB + ANB	35	59.9	22 (62.9)	Intraoperative
Huang et al, 2024^13^	Single-center RCT	61	Taiwan	US-guided SSNB + axillary nerve block (ANB)	30	64.0	16 (53.3)	Intraoperative
H Kim et al, 2021^15^	Single-center PCS	76	Korea	AS-guided continuous SSNB	38	64.8	20 (52.6)	MRI
JY Kim et al, 2021^16^	Single-center RCT	93	Korea	AS-guided continuous SSNB	31	62.4	17 (54.8)	MRI
Ko et al, 2017^17^	Single-center RCT	40	Korea	(1) AS-guided SSNB + ANB (2) Landmark-guided SSNB	(1) 20 (2) 20	(1) 56.0 (2) 56.9	(1) 6 30) (2) 7 (35)	MRI
Koga et al, 2019^18^	Single-center RCS	97	Japan	Landmark-based SSNB + GHJ injection	33	66.6	N/A	MRI
Lee et al, 2014^24^	Single-center RCT	42	Korea	(1) US-guided SSNB + ANB (2) US-guided SSNB	(1) 21 (2) 21	(1) 54.0 (2) 55.8	(1) 7 (33.3) (2) 7 (33.3)	MRI
Lee et al, 2015^25^	Mult-center RCT	30	Korea	AS-guided SSNB	15	48.9	4 (26.7)	MRI or US
Lee et al, 2017^23^	Single-center RCT	48	Korea	AS-guided SSNB + US-guided ISB	24	57.4	12 (50)	MRI
Lee et al, 2021^22^	Single-center RCT	40	Korea	(1) US-guided SSNB + ANB + Dexmedetomidine (2) US-guided SSNB + ANB	(1)20 (2) 20	(1) 58.0 (2) 63.0	(1) 11 (55) (2) 10 (50)	MRI
Lievano et al, 2023^27^	Single-center RCS	175	Columbia	(1) US-guided ISB + landmark-guided SSNB (2) Landmark-guided SSNB	(1) 61 (2) 101	(1) 58 (2) 59	(1) 30 (49) (2) 56 (55)	NA
Mittimanj et al, 2022^28^	Single-center RCS	44	India	Landmark-guided SSNB	22	59	9 (40.9)	MRI
Neuts et al, 2018^29^	Single-center RCT	100	Belgium	US-guided SSNB + ANB	48	51	30 (62.5)	NA
Park et al, 2016^30^	Single-center RCT	114	Korea	(1) Landmark-guided SSNB (2) Landmark-guided SSNB + ANB	(1) 36 (2) 37	(1) 59.2 (2) 63.3	(1) 17 (47.2) (2) 20 (52.6)	MRI
Sethi et al, 2019^33^	Single-center RCT	80	USA	Landmark based SSNB + ISB	25	56.2	12 (48)	MRI
Teratani et al, 2020^39^	Single-center RCT	128	Japan	(1) Preop US-guided SSNB + postop landmark-guided SSNB + GHJ injection + SAB injection + deltoid injections (cocktail therapy) (2) Preop US-guided SSNB + postop landmark-guided SSNB + GHJ injection + SAB injection + deltoid injections (control)	(1) 64 (2) 64	(1) 64 (2) 65.2	(1) 25 (39.1) (2) 23 (35.9)	MRI
Yamakado et al, 2014^42^	Single-center RCT	40	Japan	AS-guided SSNB	21	66	7 (33)	CT

*N/A*, not mentioned in study; *PCS*, prospective cohort study; *RCS*, retrospective cohort study; *RCT*, randomized controlled trial; *US*, ultrasound; *AS*-*guided*, arthroscopically guided; *GHJ*, glenohumeral joint; *SSNB*, suprascapular nerve block; *SAB*, subacromial bursa; *ISB*, interscalene block; *MRI*, magnetic resonance imaging; *CT*, computed tomography.

## Nonoperative suprascapular nerve block

Five studies evaluated nonoperative SSNB in patients with chronic shoulder pain, including rotator cuff-related pathology, investigating image-guided and landmark-guided techniques.^6^^,^^34^^,^^35^^,^^40^^,^^43^ All 5 were RCTs and rated as Level 2 evidence. Three studies were conducted in Australia,^6^^,^^34^^,^^35^ 1 in the United Kingdom,^40^ and 1 in Turkey.^43^ Four were single-center studies,^6^^,^^35^^,^^40^^,^^43^ while 1 was multi-center^34^ (Table I).

Two studies focused specifically on patients with confirmed rotator cuff pathology,^6^^,^^40^ while the remaining 3 included mixed populations with chronic shoulder pain but predominantly patients with clinical features suggestive of rotator cuff tears.^34^^,^^35^^,^^43^ Across all studies, a total of 300 patients (336 shoulders) were included, with female subject predominance of approximately 53%. Mean participant ages ranged from 57 to 76 years. Most participants had chronic shoulder pain of at least several months' duration prior to SSNB.

Rotator cuff pathology was confirmed using a variety of diagnostic modalities: 2 studies used magnetic resonance imaging to distinguish partial from full-thickness tears,^6^^,^^43^ 2 used ultrasound,^34^^,^^35^ and 1 used arthrography.^40^ All SSNBs contained local anesthetic (eg, ropivacaine), with corticosteroids (eg, betamethasone) used as adjuvants.

Three studies evaluated landmark-guided SSNB using consistent techniques that targeted the suprascapular notch based on bony landmarks.^34^^,^^35^^,^^40^ Both Shanahan studies used the technique described by Dangoisse et al, highlighting a common approach to anatomical localization.^7^^,^^34^^,^^35^ This technique involved drawing a line along the spine of the scapula, which was then intersected by a vertical line from the inferior angle of the scapula, effectively dividing the scapular surface into quadrants (see Supplementary Figure S1).^34^^,^^35^^,^^40^ A 21G or 22G needle was inserted 2-3 cm superior and lateral to the midpoint of this intersection. From there, the needle is advanced in the plane of the scapular blade, directing it over the spine until it contacted the floor of the supraspinous fossa.^35^ Vecchio et al used a modified approach, where the patient placed their ipsilateral hand on the opposite shoulder, elevating the scapula away from the thoracic wall, thought to reduce the risk of pneumothorax.^40^

Two studies evaluated ultrasound-guided techniques, differing in approach: one used the Harmon and Hearty method to target the suprascapular notch, while the other targeted the distal suprascapular nerve.^6^^,^^43^ Shanahan et al directly compared CT-guided SSNB with landmark-guided SSNB.^35^ The CT-guided technique targeted the suprascapular notch.^35^

### Efficacy (overall pain and functional improvement)

Across the 5 included studies evaluating SSNB for nonoperative management of chronic shoulder pain due to rotator cuff pathology, both image-guided and landmark-guided techniques demonstrated significant reductions in pain and improvements in shoulder function from baseline. Pain was assessed using various tools, including VAS, Pain Pressure Threshold, and SPADI (Table II and Table III).

**Table II:** Summary of reported reduction in pain score across included nonoperative studies.

Reference	Type of SSNB	Pain score(s)	Mean difference in pain scores (SD)	Follow-up
Coory et al, 2019^6^	US-guided SSNB	VAS (10-point scale)	NA	12 weeks
Shanahan et al, 2003^34^	Landmark-guided SSNB	VAS (100-point scale)	*VAS at rest (100)* Week 1: 14.8 (19.4) Week 4: 14.5 (22.6) Week 12: 12.9 (23.9) *VAS at night (100)* Week 1: 21.6 (25.3) Week 4: 22.4 (28.0) Week 12: 16.3 (30.5) *Pain on movement (100)* Week 1: 21.3 (22.6) Week 4: 16.9 (24.4) Week 12: 19.2 (24.5)	12 weeks
Shanahan et al, 2004^35^	(1) Landmark-guided SSNB (2) CT-guided SSNB	VAS (100-point scale)	Group 1: Landmark-guided SSNB *VAS at rest* Week 1: 23.1 Week 4: 30.0 Week 12: 17.4 *VAS at night* Week 1: 20.8 0 Week 4: 22.3 Week 12: 13.2 *VAS on movement* Week 1: 11.0 Week 4: 14.2 Week 12: 9.2 Group 2: CT-guided SSNB *VAS at rest* Week 1: 27.5 Week 4: 27.1 Week 12: 19.4 *VAS at night* Week 1: 21.7 Week 4: 24.7 Week 12: 10.3 *VAS on movement* Week 1: 10.1 Week 4: 11.0 Week 12: 2.7	12 weeks
Vecchio et al, 1993^40^	Landmark-guided SSNB	VAS (10-point scale)	*VAS at night* Week 1: 5 (1.3) Week 4: 4.2 (1.2) Week 12: 2.2 (1.8) *VAS on movement* Week 1: 5.6 (0.7) Week 4: 4.8 (1.0) Week 12: 4 (0.6)	12 weeks
Yıldızhan et al, 2024^43^	(1) US-guided SSNB at spinoglenoid notch (2) US-guided SSNB at suprascapular notch	VAS (10-point scale)	Group 1: US-guided SSNB at spinoglenoid notch *VAS during activity* 1 h: 2.3 (3.2) Week 1: 2.2 (2.5) Week 4: 2.8 (2.7) Week 12: 2.9 (2.7) *VAS at night* Week 1: 3.2 (2.6) Week 4: 3.4 (2.6) Week 12:3.1 (2.7) Group 2: US-guided SSNB at suprascapular notch *VAS during activity* 1 h: 2.6 (2.0) Week 1: 2.8 (2.5) Week 4: 3.7 (2.4) Week 12: 3.5 (2.9) *VAS at night* Week 1: 3.0 (2.9) Week 4: 3.9 (2.4) Week 12:3.3 (3.2)	12 weeks

*VAS*, visual analog scale; *SSNB*, suprascapular nerve block; *SD*, standard deviation; *CT*, computed tomography; *US*, ultrasound.

**Table III:** Summary of reported functional outcomes from nonoperative studies included.

Reference	Type of SSNB	Function score(s)	Mean difference in function scores (SD)
Shanahan et al, 2003^34^	Landmark-guided SSNB	SPADI (total) SPADI (pain) SPADI (disability)	*SPADI (total)* Week 1: 16.5 (15.3) Week 4: 13.6 (18.5) Week 12: 13.5 (19.3) *SPADI (pain)* Week 1: 22.5 (18.1) Week 4: 17.0 (20.9) Week 12: 16.6 (21.7) *SPADI (disability)* Week 1: 10.6 (16.6) Week 4: 10.25 (20.1) Week 12: 10.5 (20.2)
Shanahan et al, 2004^35^	(1) Landmark-guided SSNB (2) CT-guided SSNB	SPADI (total) SPADI (pain) SPADI (disability)	Group 1: Landmark-guided SSNB *SPADI (total)* Week 1: 15.1 Week 4: 18.1 Week 12: 9.5 *SPADI (pain)* Week 1: 18.2 Week 4: 21.8 Week 12: 11.5 *SPADI (disability)* Week 1: 12.1 Week 4: 14.4 Week 12: 7.6 Group 2: CT-guided SSNB *SPADI (total)* Week 1: 13.8 Week 4: 12.6 Week 12: 7.9 *SPADI (pain)* Week 1: 16.6 Week 4: 15.3 Week 12: 9.3 *SPADI (disability)* Week 1: 11.3 Week 4: 9.9 Week 12: 6.5
Yıldızhan et al, 2024^43^	(1) US-guided SSNB at spinoglenoid notch (2) US-guided SSNB at suprascapular notch	SPADI (pain) SPADI (disability) SPADI (total)	Group 1: US-guided SSNB at spinoglenoid notch *SPADI (pain)* Week 1: 25.9 (22.2) Week 4: 29.4 (24.1) Week 12: 30.3 (26.0) *SPADI (disability)* Week 1: 20.3 (20.3) Week 4: 26.0 (22.3) Week 12: 29.6 (22.1) *SPADI (total)* Week 1: 22.1 (20.3) Week 4: 27.0 (22.2) Week 12: 29.4 (23.0) Group 2: US-guided SSNB at suprascapular notch *SPADI (pain)* Week 1: 27.5 (24.4) Week 4: 37.6 (21.7) Week 12: 35.7 (28.6) *SPADI (disability)* Week 1: 24.8 (24.5) Week 4: 35.8 (23.8) Week 12: 33.0 (29.2) *SPADI (total)* Week 1: 25.9 (23.7) Week 4: 36.6 (22.0) Week 12: 34.1 (28.1)

*SPADI*, Shoulder Pain and Disability Index; *SSNB*, suprascapular nerve block; *SD*, standard deviation; *CT*, computed tomography; *US*, ultrasound.

Shanahan et al was the only study to directly compare landmark-guided to CT-guided SSNB.^35^ Both groups experienced significant improvements in SPADI scores from baseline. When evaluating the proportion of patients achieving a >10-point improvement, 60% of patients in the landmark group vs. 44% in the CT-guided group met this threshold at 1 week (*P* > .10). At 4 weeks, 70% vs. 50% achieved the minimal clinically important difference (*P* > .10), and at 12 weeks, 50% vs. 37% (*P* > .10), respectively. These differences were not statistically significant at any time point.^35^ Yıldızhan et al compared 2 ultrasound-guided SSNB techniques and found significant reductions in SPADI and VAS scores from baseline in both groups at all follow-up points (*P* = .001).^43^ However, baseline SPADI and Pain Pressure Threshold scores did not differ significantly between groups (*P* > .05).^43^ Shanahan et al demonstrated that SSNB using bupivacaine and methylprednisolone produced significantly greater improvements in SPADI scores compared to placebo injections of saline, supporting the analgesic and functional efficacy of the active SSNB intervention.^34^

### Pain reduction and duration of relief

All studies reported a significant reduction in pain from baseline in both intervention and control groups. Studies did not outline the total duration of relief post the longest follow-up time.

Two studies specifically reported separate mean reductions in VAS pain scores during the day and night.^34^^,^^35^ In one landmark-guided SSNB study comparing the intervention to a placebo injection, pain relief peaked at 1 week, with a mean nighttime VAS reduction of 5.0 points on a 0-10 scale.^40^ However, this effect diminished over time, with the reduction decreasing to 2.2 points at 12 weeks.^40^ A similar trend was observed in movement VAS scores, which improved by 5.6 points at 1 week, then declined to 4.0 points at 12 weeks.^40^ By contrast, in the US-guided SSNB group, the pain reduction was more stable across the 12-week period. Daytime VAS reductions ranged from 2.2 to 2.9 points, while night-time reductions ranged from 3.1 to 3.4 points,^43^ suggesting a more sustained and consistent analgesic effect compared to the landmark-guided approach.

### Functional improvement

Functional improvement was similarly observed across both groups, with notable gains in SPADI and Constant Scores over follow-up periods ranging from 1 to 12 weeks. One study reported faster short-term improvement in SPADI at 1 week following ultrasound-guided injection, but this early benefit was not sustained at later time points.^43^ Other studies found no significant difference in functional improvement trajectories between guidance techniques^34^^,^^35^(Table III).

### Patient satisfaction

Patient satisfaction was inconsistently reported across the 5 included nonoperative SSNB studies. Where documented, both image-guided and landmark-guided groups reported high overall satisfaction with the procedure and its outcomes.^35^^,^^43^ In the few studies that included formal patient satisfaction metrics or qualitative feedback, there was no statistically significant difference between the 2 techniques.^35^^,^^43^ Patients generally reported satisfaction with pain relief and tolerability of the procedure, regardless of the guidance method used.

### Complications and safety

Across the 5 included studies evaluating nonoperative SSNB for chronic shoulder pain in rotator cuff pathology, complications were infrequently reported and largely minor. No studies documented serious adverse events, such as pneumothorax or prolonged sensory or motor deficits. One study reported nonserious adverse events following both landmark- and image-guided SSNB, including transient pain and bruising at the injection site, all of which resolved with local treatment.^35^ Another study investigating ultrasound-guided SSNB documented occurrences of transient motor block and vasovagal symptoms.^43^ Overall, both landmark-guided and image-guided SSNB techniques were considered safe and well tolerated, with no consistent difference in the rate or type of complications observed between groups. Image guidance was noted in some studies to theoretically reduce the risk of vascular or pleural puncture by enhancing precision, though this was not reflected in patient outcomes measures.

When considered together, the evidence suggests that both landmark- and image-guided SSNB techniques are efficacious in reducing pain and improving shoulder function in patients with rotator cuff-related shoulder pain.

### Postoperative suprascapular nerve block

Twenty-five studies evaluated SSNB in managing postoperative pain following rotator cuff tear repair surgery. These studies investigated image-guided and landmark-guided techniques. Twenty studies were RCTs, rated as level 1 or 2 evidence. Two were nonrandomized prospective studies and 3 were retrospective studies, rated as level 3 evidence. Ten were conducted in South Korea, 3 in Japan, 2 in USA, 1 in Taiwan, 1 in Columbia, 1 in Turkey, 1 in France, 1 in Egypt, 1 in Belgium, 1 in Chile, 1 in Turkey, 1 in Canada, and 1 in India. Twenty-four were single-center studies, while 1 was multi-center (Table I).

Twenty of the studies solely focused on patients who had rotator cuff tears recovering from surgical repair. Five studies included a mixed population of chronic shoulder pain, but the majority of patients were treated for rotator cuff tears.^1^^,^^2^^,^^9^^,^^10^^,^^29^ Across all studies, a total of 1,905 patients were included, with about 48% being female subjects. Mean participant ages ranged from 38 to 67 years.

Rotator cuff tear was confirmed in all studies using magnetic resonance imaging , often supported either radiologically or via clinical tests.

Nine studies evaluated landmark-guided SSNB, using techniques such as the Dangoisse method. In 5 of the studies, patients received isolated landmark-based SSNB.^5^^,^^8^^,^^17^^,^^18^^,^^28^ However, the four other studies did not involve isolated SSNB treated groups. In 2 of these studies, a group received SSNB combined with axillary block (ANB).^12^^,^^30^ In 2 other studies, SSNB was combined with interscalene block (ISB).^27^^,^^33^ One study directly compared landmark-guided SSNB, coupled with ANB, to isolated landmark-guided SSNB.^30^ Rotator cuff repair surgery involved general anesthesia in all studies. SSNBs contained local anesthetic (eg, ropivacaine), with corticosteroids (eg, betamethasone) used as adjuvants.

Seventeen of the studies evaluated image-guided SSNB techniques. Thirteen evaluated ultrasound-guided SSNB. Six of the studies evaluated isolated SSNB, with single SSNB injections in four of the studies^2^^,^^9^^,^^11^^,^^24^ and a continuous SSNB in the other 2.^10^^,^^15^ Five of the studies investigated SSNB combined with ANB.^9^^,^^13^^,^^17^^,^^24^^,^^29^ One study evaluated SSNB combined with ISB. One study evaluated SSNB in context of a cocktail therapy, where SSNB was combined with glenohumeral joint, subacromial, and deltoid injections.^39^ One study evaluated SSNB combined with costoclavicular block, and another evaluated SSNB combined with infraclavicular block.^1^^,^^4^ Four studies evaluated arthroscopically-guided SSNB. Typically this was conducted immediately following rotator cuff repair.^15^^,^^23^^,^^25^^,^^42^ The suprascapular nerve was visualized intraoperatively through the suprascapular notch, and local anesthetic was injected directly under direct visualization.^15^^,^^23^^,^^25^^,^^42^ In all studies, patients were provided access to postoperative patient-controlled analgesia.

### Efficacy (overall pain and functional improvement)

Across all 25 studies evaluating SSNB for management of pain post rotator cuff repair, both landmark-guided and image-guided techniques demonstrated meaningful reductions in pain and improved shoulder function. Numerous tools were used to assess pain, including VAS, Numeric Rating Scale , movement evoked pain, and patient consumption of postoperative rescue analgesia. Functional outcome was assessed using many different measures: the Constant Score, Oxford Shoulder Score, and Single Numeric Assessment Evaluation score (Tables II and III).

### Visual analog scale

All studies reported a significant reduction in pain from baseline within both landmark-guided and image-guided SSNB groups. Furthermore, in both groups, pain reduction was improved at increasing time points from 1 hour to 48 hours postoperatively. Mean pain reductions from baseline typically ranged from 3.2 to 5.5 points on a VAS score on a 0-10 scale at 48 hours postoperatively. There was no significant difference between landmark- and image-guided SSNB. Of the studies reporting mean improvement in VAS, 3 evaluated isolated SSNB, with 1 landmark-based,^17^ 1 ultrasound-guided,^24^ and 1 arthroscopically-guided.^15^ In contrast, the other 5 studies reporting mean VAS changes involved SSNB combined with additional ANB, ISB, or dexmedetomidine.^12^^,^^17^^,^^22^, ^23^, ^24^

Two studies investigating ultrasound-guided SSNB had follow-up periods past 48 hours, with both finding significant pain reduction even up to 3 months postoperatively.^11^^,^^13^ No landmark-guided SSNB study had follow-up past 7 days,^18^ so the long-term efficacy of landmark-guided approaches for shoulder pain in rotator cuff tears is relatively unknown.

In the one study directly comparing landmark-guided SSNB to arthroscopic SSNB, the landmark-guided group showed significantly lower pain postoperatively.^17^ However, this is likely attributed to the additional ANB in the landmark-guided SSNB treated group.^17^

### Functional improvement

When comparing isolated landmark-guided to image-guided SSNB, the 2 injection approaches both led to significantly improved functional outcomes at the different postoperative time points assessed. However, the difference in outcome measures used in the different studies complicates direct comparisons.

### Patient satisfaction

Patient satisfaction was inconsistently reported across the 13 included postoperative SSNB studies. Where reported, both landmark-guided and image-guided groups reported high overall satisfaction with the procedure and outcomes.^1^^,^^2^^,^^9^^,^^12^^,^^17^^,^^22^^,^^23^^,^^29^ In the few cases where these metrics were reported, there was no significant difference between image-guided SSNB and landmark-guided SSNB. The duration of relief could not be directly assessed as patient outcome measures were not reported post the latest follow-up times investigated in the studies

### Complications and safety

Across the 25 studies, evaluating postoperative SSNB for rotator cuff tear repairs, complications were largely minor when reported, such as slight pain at the injection site and nausea. No study reported major adverse events.

One study assessing continuous arthroscopically-guided SSNB reported cases of neurological symptoms (slight sensory and motor deficits) and a case of partial hemi-diaphragmatic paresis.^16^ However, these symptoms quickly resolved following removal of the indwelling catheter. The pain reduction and functional outcomes using the continuous image-guided SSNB were comparable to the single-injection approaches used for both image- and landmark-guided SSNB. Given the increased risk of complications, continuous SSNB may not be necessary.

Overall, both landmark-guided and image-guided SSNB techniques were considered safe and well tolerated, with no consistent difference in the rate or type of complications observed between the 2 groups.

### Risk of bias assessment

#### Nonoperative arm (Cochrane risk of bias 2)

**Figure 2:**
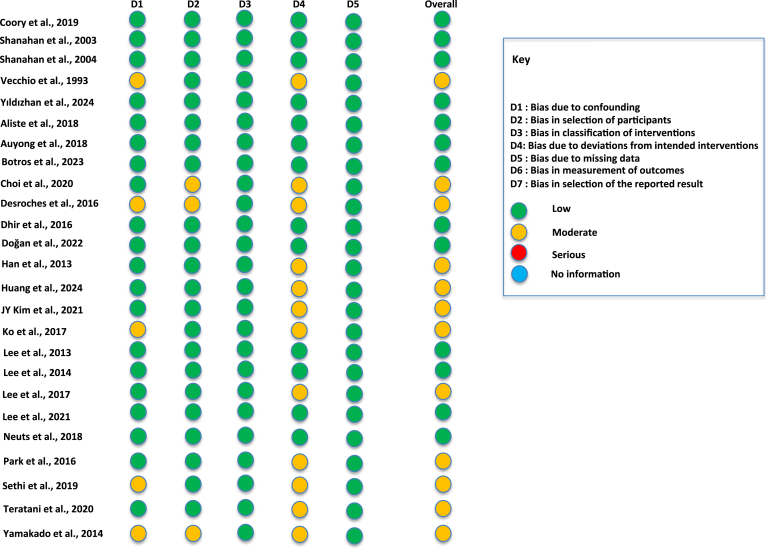
Risk of bias assessment for randomized controlled trial included with Cochrane RoB 2 assessment tool. *RoB*, risk of bias.

Among the 5 RCTs included in the nonoperative SSNB arm of this review, the RoB was generally low across all evaluated domains. Specifically, bias arising from the randomization process (D1), deviations from intended interventions (D2), missing outcome data (D3), measurement of outcomes (D4), and selection of the reported result (D5) were consistently well managed, with all studies rated as having a ‘low risk’ of bias in these areas. Accordingly, the overall RoB for studies in this arm was considered to be low (Fig. 2).

### Postoperative arm

#### Cochrane risk of bias 2 (randomized controlled trials)

In the postoperative SSNB arm, the RoBdemonstrated greater variability across domains. Bias arising from the randomization process (D1), deviations from intended interventions (D2), missing outcome data (D3), and selection of the reported result (D5) were generally well addressed, with a substantial proportion of studies rated as ‘low risk’ in these domains. However, bias in the measurement of outcomes (D4) showed more inconsistency, with several studies receiving ratings of ‘some concerns,’ particularly in cases where outcome assessors were not blinded to treatment allocation, potentially introducing detection bias. Overall, the RoBamong these RCTs ranged from low to moderate (Fig. 2).

#### Risk Of Bias In Nonrandomized Studies I (nonrandomized studies)

**Figure 3:**
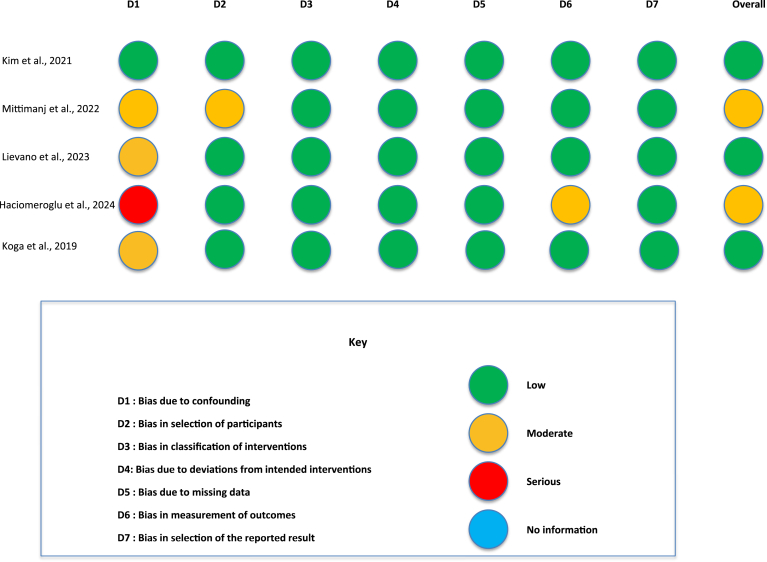
Risk of bias assessment for nonrandomized controlled trial included with ROBINS I assessment tool. *ROBINS*, Risk Of Bias In Nonrandomized Studies.

Nonrandomized studies were evaluated using the ROBINS-I tool, which examines 7 domains of bias. The most prominent concerns were observed in bias due to confounding (D1), reflecting the inherent limitations of observational designs. Despite attempts to adjust for known confounders, residual confounding could not be excluded. For instance, Haciomeroglu et al received a ‘serious’ risk rating in this domain due to inadequate adjustment for baseline differences.^11^ In contrast, domains such as bias due to classification of interventions (D3), bias due to deviations from intended interventions (D4), bias due to missing data (D5), and bias due to selection of the reported result (D7) demonstrated stronger methodological quality, with all studies achieving ‘low’ risk ratings. The overall RoBamong non-RCTs in this review ranged from moderate to low (Fig. 3).

## Discussion

In this systematic review of patients with rotator cuff tear–related shoulder pain, we found that image guidance, including US, CT, or arthroscopic assistance, does not consistently improve clinical outcomes of SSNB compared to a well-executed landmark-guided technique. Across multiple studies, there was no clear evidence of superiority in pain relief, functional improvement, complication rates, or duration of analgesia between the image-guided and landmark-guided approaches. Both techniques consistently achieved clinically significant pain reductions, along with functional gains in the short term, reinforcing the role of SSNB as an effective intervention for managing chronic shoulder pain due to rotator cuff pathology.^35^^,^^43^

Despite its theoretical advantages, the superiority of image-guidance in terms of patient-reported outcome measures was not supported by the data. This aligns with prior findings.^3^^,^^38^ Batten et al found that for mixed shoulder conditions, including rotator cuff disease, image guidance did not show a clear outcome advantage over landmark technique in their meta-analysis.^3^ This is further supported by Smith et al, who found that despite increased adoption, ultrasound-guided SSNB offered no clinical benefit over landmark-guided SSNB for chronic shoulder pain. However, this review did not perform subgroup analysis for rotator cuff tears and highlighted the current lack of clinical trials focused on this subgroup.^40^

The suprascapular nerve is typically blocked at the suprascapular notch, where local anesthetic can diffuse adequately even when not placed precisely, particularly when larger volumes are used. Studies such as Shanahan et al demonstrated equivalent pain relief between CT-guided and landmark-guided SSNB, with the landmark group using higher injectate volume, potentially compensating for imprecision.^34^ Moreover, many studies ensured that landmark injections were performed by experienced clinicians, likely reducing the procedural error.^21^^,^^32^

While outcome equivalence was observed, image-guidance offers procedural advantages such as fewer needle redirections, better first attempt success, and the ability to visualize anatomical anomalies such as variant suprascapular notches.^37^ US can also reduce the risk of inadvertent injury to adjacent structures like the pleura or suprascapular artery.^37^ Cadaveric studies confirm that image-guided injections have higher anatomic accuracy.^19^ However, clinical outcomes plateau once a certain level of accuracy is reached, diminishing the return on added precision.

Both techniques generally offered intermediate-duration relief (3 months). Recurrence is expected as neither technique alters the underlying rotator cuff pathology. It is conceivable that more accurate placement might prolong relief by targeting the nerve more precisely, but our data did not support this hypothesis.^34^^,^^35^^,^^43^

Our findings suggest that a well-performed landmark-guided SSNB is as effective as an image-guided SSNB in patients with rotator cuff tear–related pain. This is important in settings or clinics lacking ultrasound access. Saglam et al concluded that landmark-guided SSNB is a valid and effective alternative when imaging is unavailable, and this is supported by our findings.^32^ Patients should not be denied SSNB due to lack of imaging, provided it is performed safely.

No major complications were reported. While landmark SSNB theoretically carries a small risk of pneumothorax, especially with deep advancement near the suprascapular notch, this was not observed in any of our included studies.^34^^,^^35^ Image guidance could further mitigate such rare risks by visualizing the pleura and adjacent vasculature.^35^ However, in skilled hands, both techniques appear practically safe.

A subtle benefit of ultrasound-guidance is that it may allow for lower injectate volumes, which could be helpful in patients for whom local anesthetic minimization is desirable. One RCT reported no significant difference in outcomes with 70% less injectate under ultrasound, suggesting this could be a clinically meaningful optimization, although systemic and long-term effects were not measured.^35^ Some patients may appreciate the perceived precision and reassurance associated with imaging. However, this did not translate into measurably higher satisfaction scores in a consistent or quantifiable manner in the studies included in this review.

This review is limited by the modest number and size of included RCTs and by heterogeneity in patient populations. Some studies included patients with adhesive capsulitis or glenohumeral arthritis in addition to rotator cuff pathology, which may influence pain mechanisms.^34^^,^^35^ However, rotator cuff tears predominated in included studies, and results were consistent across trials. The included studies were methodologically heterogeneous, with some combining SSNB with additional nerve blocks and others employing varied patient-reported outcome measures. This variability limited comparability and a formal meta-analysis was not possible. Long-term outcomes and repeated injections were not addressed, so potential benefits of image guidance in chronic management remain unproven. In addition, patient satisfaction was not statistically assessed, though these may influence treatment preference.

Further research could focus on specific subgroups, such as *patients with difficult anatomy* (obese habitus or variant scapular anatomy); perhaps in these, ultrasound might show a benefit. In addition, considering cost-effectiveness would be valuable given that image guidance and trained personnel add cost. If outcomes are similar, one might reserve ultrasound for cases where landmark attempts failed or where risk factors are present. Another area is combining SSNB with other interventions. For instance, ultrasound allows a combination of SSNB with other injections (such as into the subacromial bursa) in one session. This was outside our scope but could be a practical advantage of imaging (guiding multi-structure injections). Lastly, patient preference can be considered. Some patients may feel more reassured knowing an imaging device is being used, potentially improving their subjective experience. None of the studies measured this, but it could be a factor in patient satisfaction.

## Conclusion

This systematic review has demonstrated that both image-guided and landmark-guided SSNB can effectively reduce pain and improve shoulder function in patients with rotator cuff tear–related shoulder pain, whether managed conservatively or postoperatively. Despite theoretical advantages regarding accuracy and safety with image guidance, these findings indicate no clear evidence to support its superiority in clinical outcomes such as pain relief, functional improvement, complication rates, or duration of analgesia when compared to landmark-guided techniques. The available evidence suggests that a carefully executed landmark-guided SSNB provides comparable clinical benefits and remains an effective alternative, especially in resource-limited settings. Image guidance may offer minor procedural advantages, such as reduced needle repositioning, improved first-attempt success rates, and potentially greater reassurance for patients. However, these advantages do not consistently translate into measurable improvements in patient satisfaction or clinical outcomes.

## Disclaimers:

Funding: No outside funding or grants were received in support of this study. The design, data extraction, analysis, and manuscript preparation were conducted independently by the authors without involvement from any external organisation or commercial sponsor.

Conflicts of interest: The authors, their immediate families, and any research foundation with which they are affiliated have not received any financial payments or other benefits from any commercial entity related to the subject of this article.
