# Supplementary material for: Image-guided versus landmark-guided suprascapular nerve block for shoulder pain in rotator cuff tears: a systematic review
Source: JSES Rev Rep Tech. 2025 Sep 17;6(1):100583. doi: 10.1016/j.xrrt.2025.09.002 (PMC12553052; doi:10.1016/j.xrrt.2025.09.002)
Supplement: Supplementary Figure 1 [file mmc1.pptx]

## Slide 1
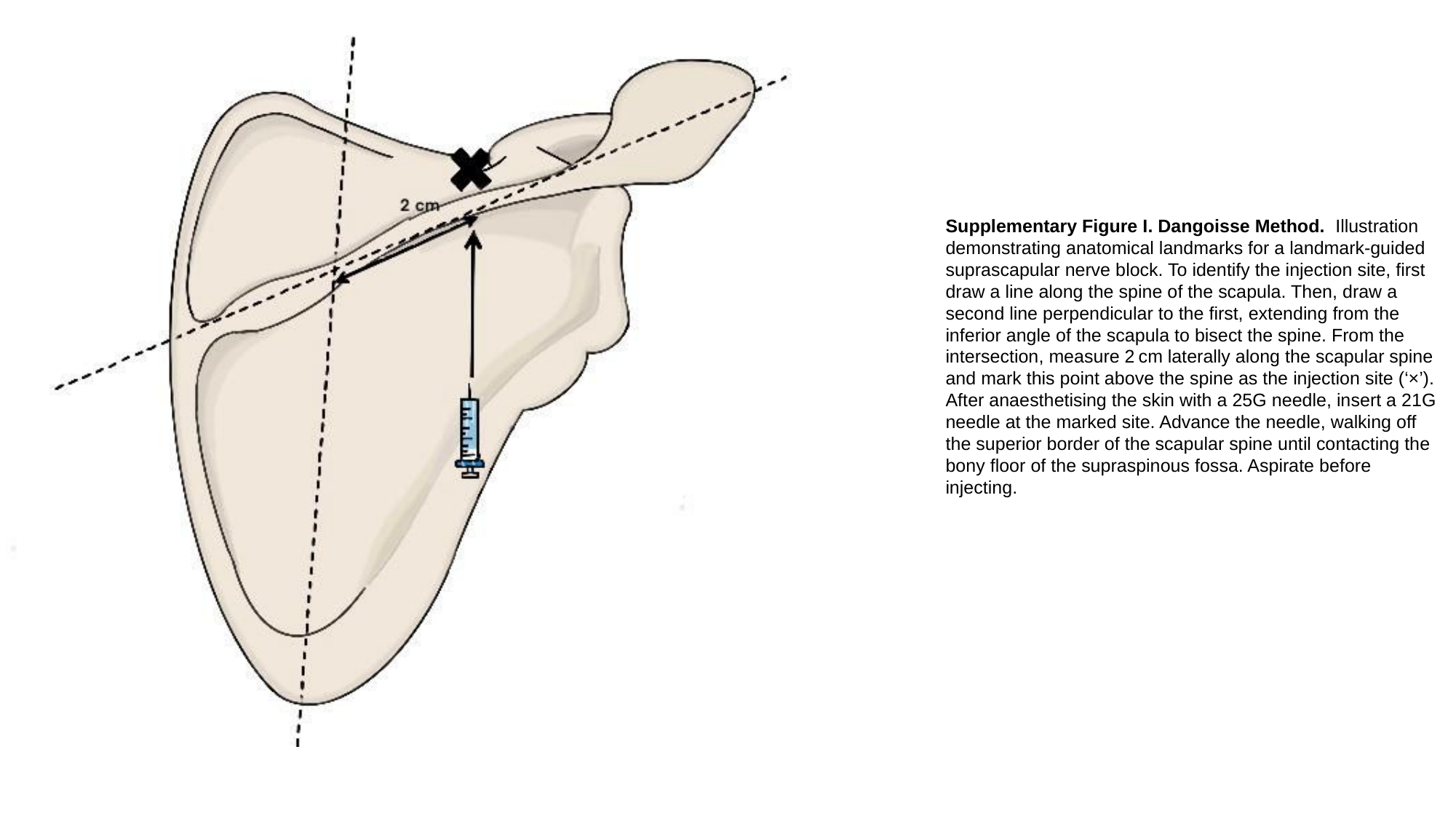

Supplementary Figure I. Dangoisse Method.  Illustration demonstrating anatomical landmarks for a landmark-guided suprascapular nerve block. To identify the injection site, first draw a line along the spine of the scapula. Then, draw a second line perpendicular to the first, extending from the inferior angle of the scapula to bisect the spine. From the intersection, measure 2 cm laterally along the scapular spine and mark this point above the spine as the injection site (‘×’). After anaesthetising the skin with a 25G needle, insert a 21G needle at the marked site. Advance the needle, walking off the superior border of the scapular spine until contacting the bony floor of the supraspinous fossa. Aspirate before injecting.
